# Effect of ramosetron on QTc interval: a randomised controlled trial in patients undergoing off-pump coronary artery bypass surgery

**DOI:** 10.1186/s12871-016-0222-1

**Published:** 2016-08-03

**Authors:** Tae Kyong Kim, Youn Joung Cho, Chae-won Lim, Jeong Jin Min, Eue-Keun Choi, Deok Man Hong, Yunseok Jeon

**Affiliations:** 1 Department of Anaesthesiology and Pain medicine, Seoul National University Hospital, Daehakro 101, Jongno-gu, Seoul 110-744 Korea; 2 Department of Anaesthesiology and Pain Medicine, Cheorwon Gil Hospital, Gangwon-Do, Korea; 3 Department of Anaesthesiology and Pain Medicine, Samsung Medical Centre, Seoul, Korea; 4 Department of Internal Medicine, Seoul National University Hospital, Seoul, Korea

**Keywords:** Cardiac surgery, Corrected QT interval, Ramosetron, Torsadogenic action, Serotonin 5-HT3 receptor antagonists

## Abstract

**Background:**

Ramosetron is a relatively new 5-hydroxytryptamine three receptor antagonist with higher binding affinity and more prolonged duration of action compared to ondansetron. The present study was performed to evaluate the effects of ramosetron on QTc interval and possible cardiovascular adverse effects in patients undergoing cardiac surgery.

**Method:**

A total of 114 patients who underwent off-pump coronary artery bypass surgery were enrolled in this randomised placebo-controlled trial. Patients were allocated into two groups that received intravenous injection of 0.3 mg ramosetron or normal saline during induction of anaesthesia. QTc intervals were measured before the operation, intraoperatively (0, 1, 2, 3, 5, 10, 15, 30, 45, 60, 90, 120, and 240 min after injection of ramosetron or normal saline), at the end of the operation, and on postoperative day 1.

**Results:**

There were no differences in mean QTc interval between groups at every time point. However, maximal change in QTc interval during surgery was higher in the ramosetron group than the placebo group (25.1 ± 22.0 vs. 17.5 ± 14.5 ms, 95 % CI 0.34–14.78, *P* = 0.040). Also, there were more patients with a QTc interval increase of > 60 ms in the ramosetron group (5 vs. 0, 95 % CI 1.6–18.0, *P* = 0.021). There were no significant differences in cardiovascular complications.

**Conclusions:**

Ramosetron administered during induction of anaesthesia may affect maximal change in QTc interval during off-pump coronary artery bypass surgery. Ramosetron should be used with caution in high risk patients for developing Torsades de Pointes.

**Trial registration:**

ClinicalTrials.gov NCT02139241. Registered November 12, 2013

**Electronic supplementary material:**

The online version of this article (doi:10.1186/s12871-016-0222-1) contains supplementary material, which is available to authorized users.

## Backgroud

5-Hydroxytryptamine 3 (5-HT_3_) receptor antagonists are widely used antiemetics for postoperative nausea and vomiting (PONV). However, QTc interval prolongation has been observed in a number of patients after administration of 5-HT_3_ receptor antagonists, such as ondansetron [1, 2]. QTc interval prolongation increases the likelihood of polymorphous ventricular arrhythmia or Torsades de Pointes (TdP), which may progress to fatal ventricular fibrillation and sudden death [3]. Previous studies showed that QTc interval prolongation is associated with increased risk of cardiovascular mortality and sudden cardiac death [4–6].

PONV is not a rare occurrence in cardiac surgery patients, with incidence rates of 35 to 71 % [7]. Long duration of anaesthesia, postoperative pain, and high levels of opioid use may contribute to PONV in cardiac surgery [8]. 5-HT_3_ receptor antagonists have been used in various clinical settings, including cardiac surgery. However, patients with organic heart disease are likely to have QTc interval prolongation [9], thus 5-HT_3_ receptor antagonist-associated prolongation of QTc interval in high-risk cardiac surgical patients may further increase QTc interval and may result in severe cardiac complications.

Ramosetron is relatively new 5-HT_3_ receptor antagonist, which has higher binding affinity and prolonged duration of action compared with ondansetron [10]. A recent meta-analysis showed that ramosetron is effective for preventing PONV and reduces the incidence of PONV compared with ondansetron [11]. However, limited data are available regarding whether ramosetron increases QTc interval. As 5-HT_3_ receptor antagonists share a mechanism of action, we hypothesised that ramosetron would be associated with QTc interval prolongation in patients undergoing cardiac surgery. To evaluate our hypothesis, we conducted a prospective, randomised, double-blind, placebo-controlled study with evaluation of QTc interval and cardiovascular adverse outcomes by ramosetron in patients undergoing off-pump coronary artery bypass graft (OPCAB).

## Methods

### Patients

This trial was registered at clinicalTrials.gov (NCT02139241) and adhered to CONSORT guidelines. The study complied with the Declaration of Helsinki and each patient gave his or her written consent to participate. There were no important changes to methods or outcomes after trial commencement and no interim analyses were performed. Adult patients scheduled for elective OPCAB were eligible for inclusion. Patients were excluded if they had preoperative treatment with inotropic agents or mechanical assist devices, heart failure with left ventricular ejection fraction less than 30 %, QTc interval prolongation of more than 500 ms on preoperative ECG, significant arrhythmia including atrial fibrillation or atrioventricular block, age more than 80 years, a history of hepatic failure (Child Class B or C), emergency operation, renal impairment requiring renal replacement therapy, history of allergy to 5-HT_3_ antagonists, recent exposure to medications known to cause QTc prolongation, or undergone concomitant major surgeries including general surgery, neurosurgery and orthopaedic surgery.

### Study design and treatments

The study was double-blind, placebo-controlled, parallel-group study conducted in Seoul National University Hospital, a tertiary hospital in Seoul, Korea. Patients were randomly allocated to either ramosetron group or placebo group, using a computer-generated random number table. A randomisation sequence was created with a 1:1 allocation using random block sise of 4. An independent nurse who was not involved in the collection of data and patient care handled the random list. All patients, medical personnel, and investigators were blinded to the allocation. One researcher (D.M.H.) generated the random allocation sequence, enrolled participants and assigned participants to interventions. The independent nurse prepared 0.3 mg of ramosetron (Nasea®; Astellas, Tokyo, Japan), the manufacturer’s recommended dose or, for the control group, the same volume of normal saline.

These two medications of the same color and volume were indistinguishable to the anaesthesiologists in charge of anaesthesia. The ramosetron group received intravenous ramosetron immediately before induction of general anaesthesia, while the placebo group received intravenous normal saline according to the same schedule.

### Anaesthesia

All patients received standard perioperative care. Routine monitoring included 5-lead ECG, pulse oximetry, non-invasive blood pressure, bispectral index, cerebral oximetry, continuous arterial blood pressure, pulmonary artery catheter, and transoesophageal echocardiography. A radial arterial catheter was put in place under local anaesthesia with lidocaine. Anaesthesia was induced with intravenous midazolam 0.15 mg/kg, sufentanil 1 μg/kg, and vecuronium 0.15 mg/kg, and maintained with continuous infusions of remifentanil 0.5–1.0 μg/kg/min and propofol 0.04–0.07 mg/kg/min, with targeting bispectral index values between 40 and 60. We did not use volatile anaesthetics to avoid their effects on QTc interval [12–14]. Arterial systolic, diastolic, and mean blood pressure, heart rate, and doses of inotropic or anticholinergic drugs were recorded at the time of induction and during the surgery. Anaesthesia-related hypotension (mean blood pressure < 60 mmHg) was treated with either ephedrine 0.1 mg/kg IBW (ideal body weight) (heart rate < 70 beats/min) or phenylephrine 0.5 μg/kg IBW (heart rate ≥ 70 beats/min). If blood pressure was not restored within 30 s, the regimen was repeated until the maximum dose of ephedrine 0.5 mg/kg IBW or phenylephrine 4 μg/kg IBW was reached. If the blood pressure was not restored by the maximum dose, vasopressin or epinephrine was administered at the anaesthesiologist’s discretion. Normothermia was maintained during the surgery with a heating mattress, warmed intravenous fluids, and a warm operating room temperature.

### QTc interval measurement and analysis

Digital ECGs were recorded using a continuous monitoring ECG system (Solar® 8000 M, GE Medical Systems, Milwaukee, WI, USA) at the beginning of drug administration, after 1, 2, 3, 5, 10, 15, 30, 45, 60, 90, 120, and 240 min, and at the end of the operation. ECG data in lead II were extracted with an analogue-to-digital converter (DI-149; DATAQ Instruments Inc., Akron, OH, USA), which was connected to the analogue output of the patient monitor, and stored on a personal computer [15]. Lead placement was consistent in the tracing of ECG. Temporally aligned superimposed ECG leads were available as an optional display. The sampling rate was 1000 Hz. At first, the QT interval was measured using a computer-based data analysis system (LabChart7; ADI Instruments, Colorado Springs, CO, USA). ECG waves of the four consecutive cycles were averaged to acquire a more accurate representation of the ECG waveform. The QT interval was corrected according to Bazett’s formula to preclude interference from heart rate (QTc = QT/RR^1/2^). Additionally, QT interval was corrected using Fridericia’s formula (QTc = QT / RR^1/3^) and Hodges formula (QTc = QT + 1.75 (heart rate – 60)). An investigator (T.K.K.) blinded to the group allocations reviewed the ECG data and checked for possible artifacts. Noise or abnormal ECG rhythms were excluded from the QTc interval measurement. After the operation, patients were checked for arrhythmias, including atrial fibrillation, atrial flutter, ventricular tachycardia, ventricular fibrillation, bradycardia (heart rate < 50 beats/min), and tachycardia (heart rate > 100 beats/min). A postoperative ECG was performed to evaluate QTc interval on the morning of postoperative day 1.

### Definition of postoperative complications

The lengths of stay in the ICU and hospital were defined as the difference in days between the date of discharge and the date of surgery. Postoperative in-hospital major adverse cardiovascular and cerebral events (MACCE) were defined as a composite of death from cardiac causes, myocardial infarction, unplanned coronary revascularisation, and stroke. Myocardial infarction was defined as elevation of troponin values (>10 × 99th percentile upper reference limit) in patients with normal baseline troponin values (<99th percentile upper reference limit). In addition, new pathological Q waves, new left bundle branch block, angiographically documented new graft or new native coronary artery occlusion, or imaging evidence of new loss of viable myocardium or new regional wall motion abnormality were required. Unplanned coronary revascularisation was defined as unplanned repeat percutaneous coronary intervention or CABG. Stroke was defined as a new ischaemic or haemorrhagic cerebrovascular accident with a neurological deficit lasting > 24 h.

### Statistical analysis

The primary endpoint was maximal intraoperative change in QTc interval after administration of ramosetron or placebo. The secondary endpoints were number of patients with QTc interval > 500 ms, which is considered to increase the risk of TdP [16]; number of patients with QTc interval increase > 60 ms, which is also considered to increase the risk of TdP [17]; presence of hypotension and bradycardia during anaesthesia induction; use of vasopressors or inotropes; presence of postoperative in-hospital arrhythmia and MACCE. Our pilot study showed that maximal change in QTc interval was 15 ± 15 ms during OPCAB. Presuming that the difference of 10 ms in the QTc intervals was clinically significant, power analysis suggested that a minimum of 49 patients would be required for each group with a type 1 error of 0.05 and a power of 0.9. Considering a 15 % dropout rate, 114 patients were recruited. Comparisons of age, weight, height, body mass index, anaesthesia time, serum electrolytes, blood pressure, heart rate, preoperative and maximal change in QTc interval, and lengths of stay in the ICU and hospital were tested with Student’s t-test or Mann–Whitney U-test after testing for normality. Sex, previous medical history, use of vasopressors or inotropes, presence of prolonged QTc interval, and presence of postoperative complications were compared by the Chi-square test or Fisher’s exact test where appropriate. QTc intervals taken serially after induction, heart rate, and blood pressure were analysed using repeated measures analysis of variance for inter- and intra-group comparisons. Statistical analyses were performed using the SPSS software (ver. 21.0; SPSS Inc., Chicago, IL, USA). In all analyses, *P <* 0.05 was taken to indicate statistical significance.

## Results

### Patients’ characteristics

**Fig. 1:**
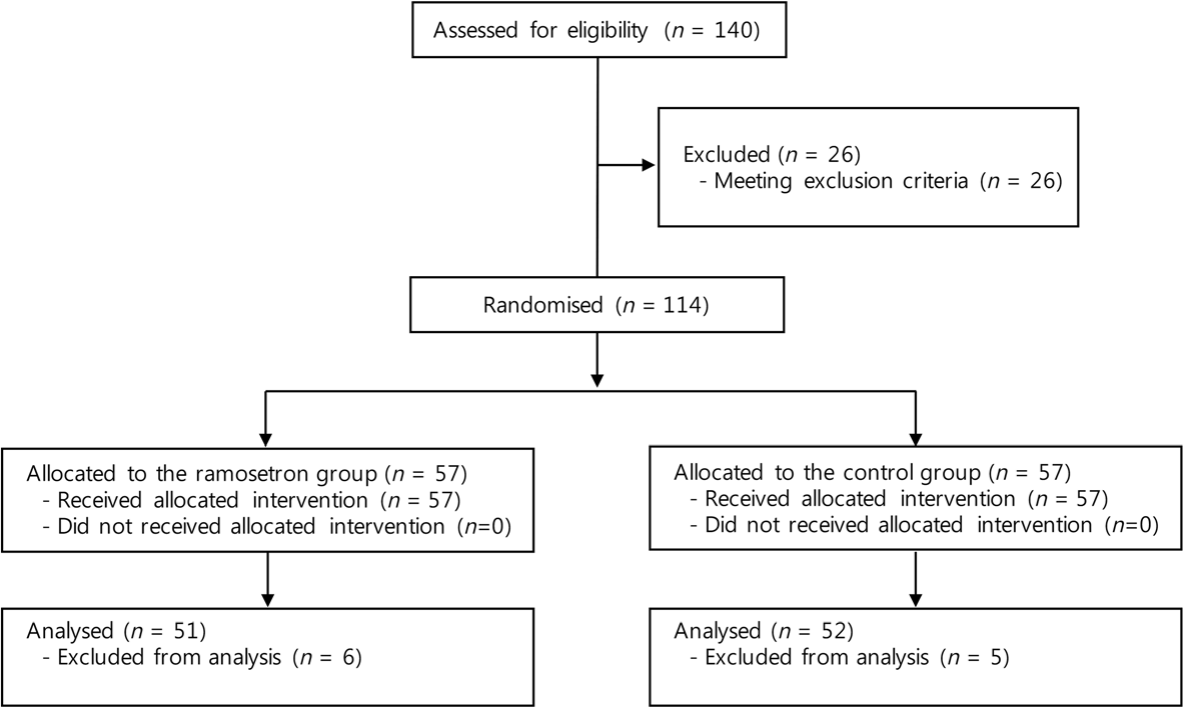
Consort diagram of study participants

A total of 140 consecutive patients treated from June 2013 to October 2014 were enrolled in this study. Of the 140 patients, 26 were excluded; 10 for left ventricular ejection fraction < 30 %, 10 for atrial fibrillation or atrioventricular block, and six for renal impairment requiring renal replacement therapy (Fig. 1). Of the 114 randomised patients, data from 11 patients could not be analysed because of poor ECG data quality or data loss. There were no cases of conversion to on-pump from OPCAB. Most surgeries began at around the same time in the morning (87.5 %); the case start time was not different between the groups. The nadir body temperature was not different between the groups (*P* = 0.106). Table 1 summarises the patients’ baseline characteristics. Preoperative bradycardia, QTc interval on ECG, and serum electrolyte levels of calcium and potassium were not significantly different between the groups.

**Table 1:** Characteristics of patients receiving ramosetron or placebo

	Ramosetron (*n* = 51)	Placebo (*n* = 52)
Male sex	42 (82.4 %)	40 (76.9 %)
Age (years)	66.8 ± 9.4	66.0 ± 9.9
Weight (kg)	66.0 ± 11.1	63.5 ± 10.3
Height (cm)	164.5 ± 9.3	162.0 ± 8.2
Body mass index (kg/m^2^)	24.4 ± 3.0	24.1 ± 3.0
Hypertension	37 (72.5 %)	33 (63.5 %)
Diabetes mellitus	22 (43.1 %)	17 (32.7 %)
1-Vessel disease	0 (0.0 %)	4 (7.7 %)
2-Vessel disease	6 (11.8 %)	8 (15.4 %)
3-Vessel disease	45 (88.2 %)	40 (76.9 %)
Previous myocardial infarction	3 (5.9 %)	4 (7.7 %)
Previous stroke	3 (5.9 %)	4 (7.7 %)
Current smoker	11 (21.6 %)	9 (17.3 %)
Sodium (mEq/l)	138.6 ± 3.7	139.1 ± 2.9
Potassium (mEq/l)	4.1 ± 0.4	4.0 ± 0.4
Bradycardia (heart rate <50 beats/min)	2 (4.0 %)	2 (3.9 %)
Left ventricle ejection fraction		
> 49 %	48 (94.1 %)	48 (92.3 %)
30–49 %	3 (5.9 %)	4 (7.7 %)
< 30 %	0 (0.0 %)	0 (0.0 %)
Preoperative QTc interval (ms)	430.7 ± 33.6	425.2 (29.7)
Preoperative QTc interval > 500 ms	0 (0.0 %)	0 (0.0 %)
Congenital long QT syndrome	0 (0.0 %)	0 (0.0 %)
Duration of anaesthesia (min)	449.7 ± 44.2	451.4 ± 68.0

Data are presented as mean ± SD or number (proportion)

### Perioperative haemodynamic parameters

**Fig. 2:**
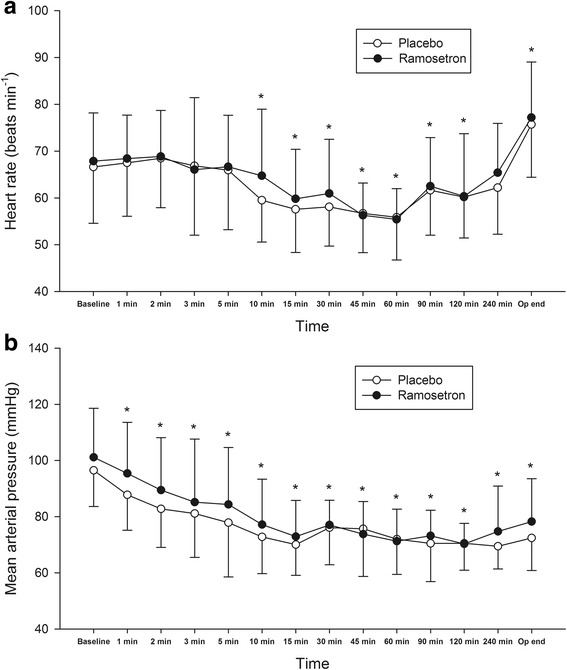
Heart rate (**a**) and mean arterial pressure (**b**) during surgery. Values are shown as means (SD). * *P* < 0.05 compared to baseline

The changes in heart rate and mean arterial pressure over time were significant in both ramosetron and placebo groups (*P <* 0.001 and *P <* 0.001, respectively) (Fig. 2), but were not different between the groups (*P =* 0.210 and *P =* 0.178, respectively). There were no intergroup differences regarding the use of ephedrine or phenylephrine during anaesthesia induction (*P =* 0.208, and 0.603, respectively), the use of inotropes or vasopressors at the end of surgery (*P =* 1.000, and 1.000, respectively), or total amount of infused fluid during the surgery (*P =* 0.666) (Table 2).

**Table 2:** Haemodynamic variables and fluids administered during surgery

	Ramosetron (*n* = 51)	Placebo (*n* = 52)	*P* value
Before anaesthesia induction			
Baseline heart rate (beats/min)	66.1 ± 10.6	68.3 ± 11.7	0.317
Baseline mean arterial pressure (mmHg)	96.3 ± 15.8	101.1 ± 14.6	0.117
During anaesthesia induction			
Use of ephedrine	37 (72.5 %)	31 (60.8 %)	0.294
Use of phenylephrine	8 (15.7 %)	10 (19.6 %)	0.796
At the end of surgery			
Use of vasopressors	1 (1.9 %)	1 (2.0 %)	1.000
Use of inotropes	1 (1.9 %)	1 (2.0 %)	1.000
Number of grafts (n)	3.6 ± 0.7	3.3 ± 1.1	0.120
Total infused fluids (ml)	3671.2 ± 1368.4	3557.1 ± 1308.9	0.666
Total infused crystalloids (ml)	3083.3 ± 1699.3	2992.5 ± 1612.8	0.781
Total infused colloids (ml)	585.9 ± 638.7	526.2 ± 814.6	0.680

Data are presented as mean ± SD or number (proportion)

### Perioperative QTc interval

**Fig. 3:**
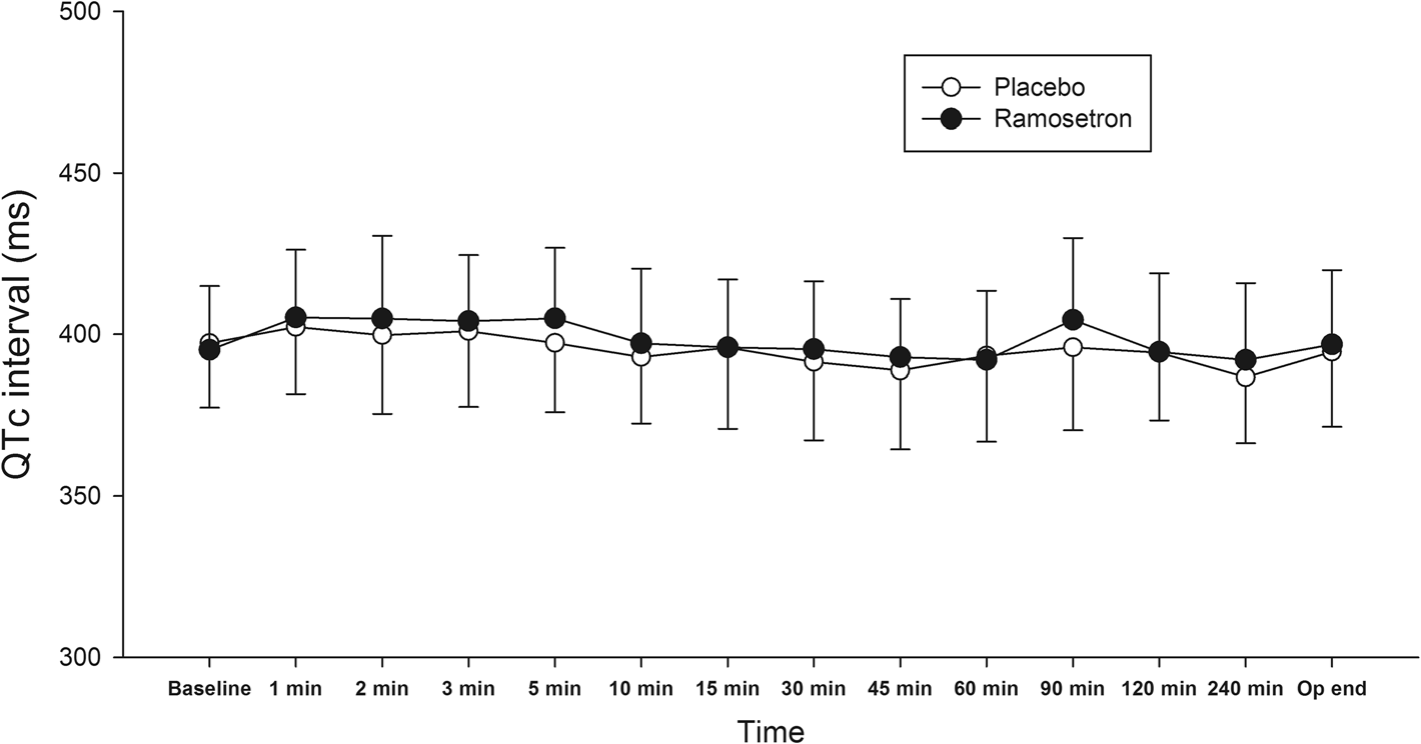
QTc interval during surgery. Values are shown as means (SD)

**Fig. 4:**
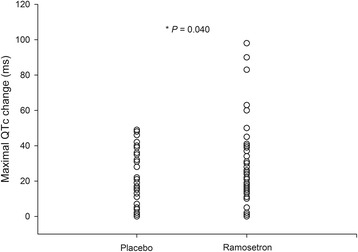
Maximal change in QTc interval during surgery. Individual data points are superimposed within each group

The changes in mean intraoperative QTc interval over time were not different between the groups (*P =* 0.591, Fig. 3). However, maximal change in QTc interval during surgery was higher in the ramosetron group than the placebo group (mean difference 7.56 ms, 95 % CI 0.34–14.78, *P =* 0.040, Fig. 4). The peak effect of ramosetron on QTc was observed at [median (IQR), 5 (2–30)] min after anaesthesia induction. Prolongation of QTc interval > 500 ms was not significantly different between groups. However, the number of patients with QTc interval increase > 60 ms was higher in the ramosetron group (risk difference 9.8 %, 95 % CI 1.6–18.0, *P =* 0.021) (Table 3). Five patients with an increase in QTc > 60 ms had baseline QTc values of 381.4 ± 36.0 ms, whereas the patients with QTc ≤ 60 ms had baseline QTc values of 397.0 ± 18.6 ms (*P* = 0.086). When applying the Hodges formula, maximal change in QTc interval was higher in the ramosetron group with a marginal significance (mean difference 8.81 ms, 95 % CI −0.99–18.61, *P* = 0.077). Also, there were more patients with a QTc interval increase of > 60 ms in the ramosetron group (risk difference 9.8 %, 95 % CI 1.6–18.0, *P* = 0.021) (Additional file 1 shows QTc interval by Fridericia’s formula and Hodges formula). None of the patients experienced clinically significant arrhythmias requiring treatment during surgery.

**Table 3:** Changes in QTc interval during the surgery

	Number of patients (%)			
	Ramosetron (*n* = 51)	Placebo (*n* = 52)	Risk difference (95 % CI)	*P* value
QTc interval prolongation > 500 ms	1 (2.0 %)	0 (0.0 %)	2.0 % (−1.8 to 5.8)	0.495
QTc interval increase > 60 ms	5 (9.8 %)	0 (0.0 %)	9.8 % (1.6 to 18.0)	0.021
QTc interval increase > 30 ms	13 (25.5 %)	12 (23.1 %)	2.4 % (−14.2 to 19.0)	0.775
	Mean (SD)		Mean difference (95 % CI)	
Baseline QTc interval before injection (ms)	395.1 ± 19.7	397.2 ± 19.8	−2.09 (−9.85 to 5.66)	0.593
Maximal change in QTc interval (ms)	25.1 ± 22.0	17.5 ± 14.5	7.56 (0.34 to 14.78)	0.040
QTc interval at postoperative day 1 (ms)	428.9 ± 59.0	437.9 ± 56.3	−9.02 (−33.32 to 15.28)	0.463

Data are presented as mean ± SD or number (proportion). CI, confidence interval

### Postoperative in-hospital complications

There were no significant differences between the groups in the incidences of postoperative arrhythmias, including atrial fibrillation, atrial flutter, ventricular tachycardia, ventricular fibrillation, bradycardia, and tachycardia (Table 4). The lengths of stay in the intensive care unit (ICU) and hospital, all components of MACCE, including death from cardiac causes, myocardial infarction, unplanned coronary revascularisation, and stroke, were not different between the groups (Table 4). However, this study was not powered to assess the effect of ramosetron on postoperative complications.

**Table 4:** Postoperative in-hospital complications

	Ramosetron (*n* = 51)	Placebo (*n* = 52)	RR (95 % CI)	*P* value
Atrial fibrillation	11 (21.6 %)	14 (26.9 %)	0.80 (0.40 to 1.60)	0.647
Atrial flutter	1 (2.0 %)	1 (1.9 %)	1.02 (0.07 to 15.87)	1.000
Ventricular tachycardia	0 (0.0 %)	0 (0.0 %)		
Ventricular fibrillation	0 (0.0 %)	0 (0.0 %)		
Bradycardia (heart rate <50 beats/min)	0 (0.0 %)	2 (3.8 %)		0.495
Tachycardia (heart rate > 100 beats/min)	5 (9.8 %)	8 (15.4 %)	0.64 (0.22 to 1.82)	0.555
Death from cardiac causes	0 (0.0 %)	0 (0.0 %)		
Myocardial infarction	0 (0.0 %)	0 (0.0 %)		
Unplanned coronary revascularisation	0 (0.0 %)	1 (1.9 %)		1.000
Stroke	1 (2.0 %)	1 (1.9 %)	1.02 (0.07 to 15.87)	1.000
Intensive care unit length of stay (day)	2 (1 to 3)	2 (1 to 4)		0.912
Hospital length of stay (day)	9 (7 to 13)	8.5 (7 to 13.75)		0.350

Data are presented as number (proportion) or median (IQR). CI, confidence interval; RR, relative risk

## Discussion

In the present study, 0.3 mg of intravenous ramosetron was associated with a significant increase of maximal change in QTc interval during OPCAB. Also, increase of the QTc interval of more than 60 ms, which is considered to increase the risk of TdP was more frequently observed in the ramosetron group. There were no statistically significant differences in the intraoperative haemodynamic variables measured.

Most QTc interval prolonging drugs act by blocking the potassium channel encoded by the human ether-a-go-go-related gene (hERG) [18]. 5-HT_3_ antagonists share the same potential proarrhythmic mechanisms. However, not all drugs in the 5-HT_3_ antagonist class have clinically significant QTc-interval-prolonging effects. Ondansetron produced dose-related prolongation of QTc interval [1, 2], but granisetron and palonosetron did not induce significant QTc interval prolongation [19, 20]. There are limited data regarding the effects of ramosetron on QTc interval in high-risk patients undergoing cardiac surgery.

Postoperative QTc interval prolongation was observed in 80 % of patients undergoing noncardiac surgery [21]. Multiple drugs, including opioids, general anaesthetics, antibiotics, and cardioactive drugs, are associated with QT prolongation. In a recent study, QTc interval prolongation was common in patients undergoing cardiothoracic surgery [22]. However, this report did not describe the method of anaesthesia including the use of volatile anaesthetics, which prolong QTc interval and confound the effects of 5-HT_3_ antagonists on QTc interval [12–14]. In the present study, general anaesthesia was maintained during surgery using continuous infusion of propofol and remifentanil, which are known to have minimal effects on QTc interval [14]. Mean intraoperative QTc intervals did not increase compared to baseline QTc value in both the ramosetron and placebo groups (Fig. 3). A previous study indicated that tracheal intubation caused significant prolongation of QTc interval during the anaesthetic induction period due to sympathetic stimulation [23]. However, in this study, tracheal intubation was performed 7.6 ± 1.6 min after administration of ramosetron or placebo, and it did not significantly prolong QTc interval (Fig. 3). Sufentanil administered during anaesthetic induction may attenuate the sympathetic stimuli during tracheal intubation [24].

A recent study concluded that mean QTc interval did not increase after ramosetron administration in adult patients undergoing laparoscopic cholecystectomy, which was consistent with our results [25]. However, they did not compare the maximal changes in QTc interval. In this observational trial, the QTc interval was measured only during the last 10 min of surgery. To the best of our knowledge, there have been no randomised placebo controlled studies to investigate the effects of ramosetron on the QTc interval throughout the surgery.

In the present study, there were no differences in mean QTc interval between the ramosetron group and placebo group at every time point (Fig. 3). There were no differences in the measured intra- or post-operative outcomes. It is possible that the effect of QTc prolongation may not be so strong to be clinically significant. However, ramosetron increased the maximal change in QTc interval during the surgery, and there were more patients with a QTc interval increase of > 60 ms in the ramosetron group (Table 3). The findings were consistent with the other calculation methods, including Fridericia’s, and Hodges formulas (Additional file 1). Given the effect of ramosetron on QTc interval, we have changed our clinical practice to avoid ramosetron in patients at high risk for developing TdP. Since intraoperative QTc interval prolongation was common, we recommend administering ramosetron at the end of surgery.

This study had several limitations. First, ramosetron was administered at the beginning of anaesthesia induction along with other drugs for induction of anaesthesia and possible drug-drug interactions may have confounded the effects of ramosetron on QTc interval. Moreover, a previous study indicated that propofol, which was continuously infused during surgery, may counteract the prolongation of QTc interval [26]. However, induction anaesthetics, including midazolam, vecuronium, and sufentanil, are known to have minimal effects on QTc interval [27, 28], and we did not use volatile anaesthetics that are known to prolong QTc interval during this study [12–14]. Second, intraoperative factors, some of which may be difficult to control for, may have affected QTc interval. Surgical stress itself may also contribute to QTc interval [29]. Third, the QTc interval varies between leads, and the most appropriate lead to measure the QTc interval has not been established.

## Conclusion

Ramosetron (0.3 mg) administered during induction of anaesthesia may increase maximal change in QTc interval during OPCAB. Ramosetron should be used with caution in high risk patients for developing TdP.

## Abbreviations

5-HT3, 5-Hydroxytryptamine 3; hERG, human ether-a-go-go-related gene; ICU, intensive care unit; MACCE, major adverse cardiovascular and cerebral events; OPCAB, off-pump coronary artery bypass graft; PONV, postoperative nausea and vomiting; TdP, torsades de pointes

## Additional file

Additional file 1: QTc interval by Fridericia’s formula and Hodges formula. (DOCX 17 kb)
