# Supplementary material for: Effect of ramosetron on QTc interval: a randomised controlled trial in patients undergoing off-pump coronary artery bypass surgery
Source: BMC Anesthesiol. 2016 Aug 3;16:56. doi: 10.1186/s12871-016-0222-1 (PMC4972982; doi:10.1186/s12871-016-0222-1)
Supplement: Additional file 1: — QTc interval by Fridericia’s formula and Hodges formula. (DOCX 17 kb) [file 12871_2016_222_MOESM1_ESM.docx]

**Table S1**. QTc interval by Fridericia’s formula

|  | Number of patients (%) | |  |  |
| --- | --- | --- | --- | --- |
|  | Ramosetron (*n* = 51) | Placebo (*n* = 52) | Risk Difference  (95% CI) | *P* value |
| QTc interval prolongation > 500 ms | 1 (2.0%) | 0 (0.0%) | 2.0% (-1.8 to 5.8) | 0.495 |
| QTc interval increase  > 60 ms | 5 (9.8%) | 0 (0.0%) | 9.8% (1.6 to 18.0) | 0.021 |
| QTc interval increase  > 30 ms | 14 (27.5%) | 11 (21.2%) | 6.3% (-10.2 to 22.8) | 0.456 |
|  | Mean (SD) | | Mean Difference (95% CI) |  |
| Baseline QTc interval before injection (ms) | 389.6 ± 20.7 | 388.9 ± 19.2 | 0.68 (-7.13 to 8.50) | 0.863 |
| Maximal change in QTc interval (ms) | 29.6 ± 24.0 | 21.2 ± 16.2 | 8.39 (-1.48 to 18.27) | 0.094 |

Data are presented as mean ± SD or number (proportion). CI, confidence interval. Fredericia’s formula, QTc = QT / RR^1/3^

**Table S2**. QTc interval by Hodges formula

|  | Number of patients (%) | |  |  |
| --- | --- | --- | --- | --- |
|  | Ramosetron (*n* = 51) | Placebo (*n* = 52) | Risk Difference  (95% CI) | *P* value |
| QTc interval prolongation > 500 ms | 1 (2.0%) | 0 (0.0%) | 2.0% (-1.8 to 5.8) | 0.495 |
| QTc interval increase  > 60 ms | 5 (9.8%) | 0 (0.0%) | 9.8% (1.6 to 18.0) | 0.021 |
| QTc interval increase  > 30 ms | 17 (33.3%) | 18 (34.6%) | -1.3% (-19.6 to 17.0) | 0.891 |
|  | Mean (SD) | | Mean Difference (95% CI) |  |
| Baseline QTc interval before injection (ms) | 388.7 ± 22.0 | 387.5 ± 18.2 | 1.21 (-6.69 to 9.10) | 0.763 |
| Maximal change in QTc interval (ms) | 31.5 ± 24.0 | 22.7 ± 17.0 | 8.81 (-0.99 to 18.61) | 0.077 |

Data are presented as mean ± SD or number (proportion). CI, confidence interval. Hodges formula, QTc = QT + 1.75 (heart rate – 60)
